# Unanchored Ubiquitin Chains, Revisited

**DOI:** 10.3389/fcell.2020.582361

**Published:** 2020-10-26

**Authors:** Jessica R. Blount, Sean L. Johnson, Sokol V. Todi

**Affiliations:** ^1^Department of Pharmacology, Wayne State University School of Medicine, Detroit, MI, United States; ^2^Department of Neurology, Wayne State University School of Medicine, Detroit, MI, United States

**Keywords:** poly-ubiquitin, cell stress, deubiquitinase, immune system, NF-κB, proteasome, ligase, protein quality control

## Abstract

The small modifier protein, ubiquitin, holds a special place in eukaryotic biology because of its myriad post-translational effects that control normal cellular processes and are implicated in various diseases. By being covalently conjugated onto other proteins, ubiquitin changes their interaction landscape - fostering new interactions as well as inhibiting others - and ultimately deciding the fate of its substrates and controlling pathways that span most cell physiology. Ubiquitin can be attached onto other proteins as a monomer or as a poly-ubiquitin chain of diverse structural topologies. Among the types of poly-ubiquitin species generated are ones detached from another substrate - comprising solely ubiquitin as their constituent - referred to as unanchored, or free chains. Considered to be toxic byproducts, these species have recently emerged to have specific physiological functions in immune pathways and during cell stress. Free chains also do not appear to be detrimental to multi-cellular organisms; they can be active members of the ubiquitination process, rather than corollary species awaiting disassembly into mono-ubiquitin. Here, we summarize past and recent studies on unanchored ubiquitin chains, paying special attention to their emerging roles as second messengers in several signaling pathways. These investigations paint complex and flexible outcomes for free ubiquitin chains, and present a revised model of unanchored poly-ubiquitin biology that is in need of additional investigation.

## An Introduction to Ubiquitin, Its Processing, and Unanchored Chains

Few proteins hold as central a place in eukaryotic cellular biology as the highly conserved, 76-residue protein, ubiquitin (Ub). Through a chemical reaction that enables its tethering onto essentially any other protein, Ub provides exceptionally flexible control over processes from cell division to cell death, from gene transcription to protein degradation, by dictating protein interaction, function, and turnover (Komander and Rape, 2012; Swatek and Komander, 2016; Oh et al., 2018). It is not surprising that, because of its elemental importance in eukaryotes, Ub and the pathways that it regulates are linked to various diseases.

**FIGURE 1:**
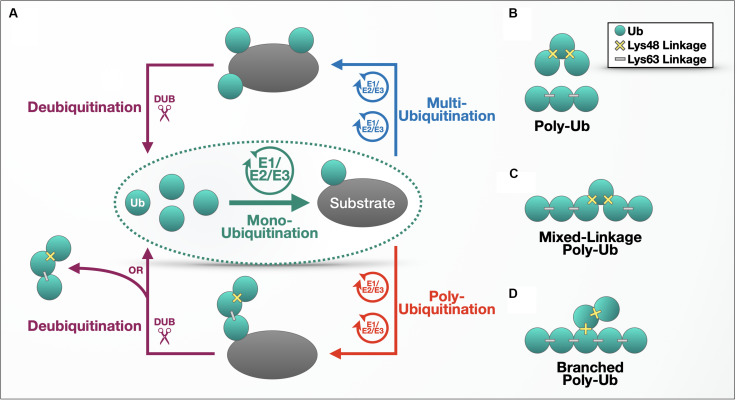
The ubiquitination process. **(A)** Outline of the ubiquitination process. **(B–D)** Different types of ubiquitin chains. Details are provided in the main text.

Ub’s modulatory properties commonly begin with the process of “ubiquitination,” which refers to the ATP-dependent conjugation of a Ub molecule onto a substrate protein via an isopeptide bond between the C-terminal carboxylic group of a Ub and the ε-amine of a lysine residue within the substrate. The cellular machinery that brings about this conjugation consists of three main components: a Ub-activating enzyme (E1), a Ub-conjugating enzyme (E2), and a Ub ligase (E3). A protein that has accepted a single Ub molecule is said to be mono-ubiquitinated; additional ubiquitination can result in a multi-ubiquitinated protein decorated with individual Ub molecules (Figure 1A). Following a mono-ubiquitination event, the conjugated Ub can also be ubiquitinated itself, forming a polymeric chain (Figures 1A,B). Each Ub harbors seven lysine residues (Lys6, 11, 27, 29, 33, 48, and 63) that themselves serve as Ub acceptor sites, spread over the surface of the protein and oriented in distinct directions. In addition, an eighth ubiquitination site exists at the N-terminal methionine (Met1) (Komander and Rape, 2012).

Poly-Ub chains are characterized by the Lys or Met residue used for chain elongation and can be composed of homogenous linkages (e.g., a tri-Ub chain with only Lys48 linkages; Figure 1B); multiple linkage types within a continuous chain (Figure 1C); or branched chains, where a Ub chain attached to a second string of poly-Ub leads to topology that resembles branches on a tree (Figure 1D). Linkage type also determines the conformation and flexibility of the chain – while Met1- and Lys63-linked chains adopt open conformations with more space between Ub molecules, Lys6-, Lys11-, and Lys48-linked poly-Ub are more compact, according to structural studies (Tenno et al., 2004; Varadan et al., 2004). The Lys48- and Lys63-linked chains diagrammed in Figure 1B illustrate the basic difference between compact and open conformations.

The shape of a Ub chain affects its binding partners, as some proteins are attracted by the pockets created by specific linkage types (Hicke et al., 2005; Dikic et al., 2009). This binding landscape gains even greater complexity through mixed-linkage and branched chains and through additional post-translational modifications to Ub, including phosphorylation (Yau and Rape, 2016; Song and Luo, 2019). Two poly-Ub chains that contain the same linkage types can behave in unique ways, depending on the arrangement of those linkages, the chains’ proximity to post-translational modifiers that act upon them, and the accessibility to Ub-binding proteins that bind specific chain types to direct them to signaling pathways or organelles (Oh et al., 2018).

The composition of a poly-Ub chain and the context of its attachment to a substrate can trigger different outcomes in the cell. (Table 1 summarizes functions of poly-Ub chains of each linkage type and enzymes reported to construct, edit, or dismantle them.) Some Ub chains target proteins to the 26S proteasome for degradation, where the poly-Ub chain is removed to be recycled whereas the protein conjugated to it is unfolded and degraded. Lys48-linked chains are the most abundant linkage with this role, although Lys11-, Lys29-, and in some cases Lys63-linked chains, have also been implicated in this functional degradation pathway (Thrower et al., 2000; Bremm and Komander, 2011; Besche et al., 2014). Branched Lys11/Lys48-linked Ub chains have also emerged as an enhanced degradation signal vital to both cell-cycle regulation and in the quality control of aggregation-prone proteins (Meyer and Rape, 2014; Yau et al., 2017). Lys48/Lys63-linked, branched chains are another degradation signal that associates with the proteasome (Ohtake et al., 2018).

**TABLE 1:** Summary of cellular processes associated with specific types of Ub linkages, and some of the E2s, E3s, and DUBs associated with them. (List not exhaustive; additional enzymes covered in main text.)

	**Reported cellular processes**	**Abundance**	**E2s and E3s reported**	**DUBs reported**
			***Bold: linkage-specific***	***Bold: linkage-specific***
Lys6	**Mitochondrial homeostasis** • Mitophagy delayed in cells with mutant Lys6 Ub (Cunningham et al., 2015; Ordureau et al., 2015) • Involved with mitochondrial homeostasis (Durcan et al., 2014; Ordureau et al., 2014, 2015; Cunningham et al., 2015) • Observed on ubiquitinated mitochondrial membrane proteins after mitochondrial depolarization (Ordureau et al., 2014) **DNA damage response** • Regulates DNA damage response with the E3 BRCA1/BARD1 (Wu-Baer et al., 2003; Morris and Solomon, 2004; Nishikawa et al., 2004)	• Does not increase with protease inhibition (Kim et al., 2011; Wagner et al., 2011) • Increases upon UV-based genotoxic stress (Elia et al., 2015) • Increases in response to mitochondrial depolarization (Ordureau et al., 2014)	**E2** • No specific E2s reported **E3** • Bacterial HECT-like E3 (Lin et al., 2011) • N1eL (Hospenthal et al., 2013) • BRCA1/BARD1 (Wu-Baer et al., 2003; Morris and Solomon, 2004; Nishikawa et al., 2004) • Parkin (Durcan et al., 2014; Ordureau et al., 2014; Cunningham et al., 2015)	• **USP30** (localized to mitochondria) (Bingol et al., 2014; Cunningham et al., 2015; Liang et al., 2015)
Lys11	**Cell cycle regulation** • Triggers proteasomal degradation of cell cycle regulators during mitosis (Jin et al., 2008; Williamson et al., 2009; Matsumoto et al., 2010; Bremm and Komander, 2011; Wickliffe et al., 2011; Castańeda et al., 2013; Meyer and Rape, 2014) **Proteasomal degradation** • Homotypic Lys11-linked chains are typically poor substrates for the proteasome (Matsumoto et al., 2010; Bremm and Komander, 2011; Wickliffe et al., 2011; Castańeda et al., 2013; Meyer and Rape, 2014; Grice et al., 2015) • The E2 UBE2S and E3 APC/C construct branched chains comprising Lys11 that are strong proteasomal degradation signals, as well as heterotypic Lys11/Lys48-linked chains (Meyer and Rape, 2014; Grice et al., 2015; Min et al., 2015) **Other** • Implicated in cell-cycle-independent processes: • Hif1(alpha) transcription factor (Bremm et al., 2014); ERAD (Xu et al., 2009b); innate immune response (Qin et al., 2014); cellular adaptation to hypoxia (Bremm et al., 2010; Moniz et al., 2015)	• Increases in response to proteasome inhibition (Xu et al., 2009b; Kim et al., 2011) • Increases in response to mitochondrial depolarization (Ordureau et al., 2014) • Preferentially produced during mitosis and G1 in cells (Xu et al., 2009a; Matsumoto et al., 2010; Meyer and Rape, 2014)	**E2** • **Ube2S** (Baboshina and Haas, 1996; Jin et al., 2008; Garnett et al., 2009; Williamson et al., 2009; Wu et al., 2010; Wickliffe et al., 2011; Min et al., 2015) • Ube2C/UbcH10 (Kirkpatrick et al., 2006; Jin et al., 2008; Bosanac et al., 2011) **E3** • **APC/C** (Baboshina and Haas, 1996; Jin et al., 2008; Garnett et al., 2009; Williamson et al., 2009; Wu et al., 2010; Min et al., 2015) • Parkin (Sarraf et al., 2013; Durcan et al., 2014; Ordureau et al., 2014; Cunningham et al., 2015) • UBR5 (Yau and Rape, 2016) • AREL1 (Kristariyanto et al., 2015b; Michel et al., 2015; Swatek and Komander, 2016)	• **OTUD7B** (Mevissen et al., 2013) • **Cezanne2/OTUD7A** (Mevissen et al., 2013) • **USP30** (Cunningham et al., 2015)
Lys27	**DNA damage response** • The E3 ligase RNF168 promotes ubiquitination of histone 2A, the major form of ubiquitination on chromatin following DNA damage (Gatti et al., 2015) • Serves as scaffolding to recruit DNA damage response mediators (Liu et al., 2014a; Gatti et al., 2015) • Lack of Lys27-linked chains prevents activation of DNA damage response (Gatti et al., 2015) **Immune response** • Associated with the host immune response in response to microbial DNA (Ishikawa et al., 2009; Wang et al., 2014b) • Lys27-linked poly-Ub of STING acts as a scaffold for the recruitment and activation of the kinase TBK1 (Ishikawa et al., 2009; Wang et al., 2014b). This association triggers a cascade that leads the activation of transcription factor IRF-3 and induction of type-1 interferons and pro-inflammatory cytokines (Ishikawa et al., 2009; Wang et al., 2014b)	• The major Ub chain type on chromatin following DNA damage (Gatti et al., 2015)	**E2** • No specific E2s reported **E3** • Parkin (Doss-Pepe et al., 2005; Geisler et al., 2010) • AMFR (Ishikawa et al., 2009; Wang et al., 2014b) • RNF168 (Gatti et al., 2015) • HACE1 (Liu et al., 2014b; Palicharla and Maddika, 2015)	• None reported
Lys29	**Proteasomal degradation** • Associates with the 26S proteasome and contributes to substrate turnover in the Ub-fusion-degradation pathway (Johnson et al., 1995; Koegl et al., 1999; You and Pickart, 2001; Besche et al., 2014) **Repression of Wnt/β-catenin signaling** • Lys29-linked poly-Ub of Axin disrupts its interaction with co-receptors and represses Wnt/β signaling (Fei et al., 2013)	• Increases upon inhibition of the proteasome (Kim et al., 2011)	**E2** • None reported **E3** • KIAA10/UBE3C (You and Pickart, 2001; Wang and Pickart, 2005; Wang et al., 2006; Kristariyanto et al., 2015a; Michel et al., 2015) • UBR5 (Yau and Rape, 2016) • UFD4 (Tsuchiya et al., 2013)	• **TRABID** (Swatek and Komander, 2016)
Lys33	**Post-Golgi membrane trafficking** • Implicated in regulating traffic through the post-Golgi network (Yuan et al., 2014) **Other** • Associated with negative regulation of T-cell antigen receptor (Huang et al., 2010) • Associated with negative regulation of AMPK-related protein kinases (Al-Hakim et al., 2008)	• Increases upon UV-based genotoxic stress (Elia et al., 2015)	**E2** • None reported **E3** • CUL3 (Yuan et al., 2014) • AREL1 (Kristariyanto et al., 2015b; Michel et al., 2015; Swatek and Komander, 2016)	• **TRABID** (Swatek and Komander, 2016)
Lys48	**Proteasomal degradation** • Targets proteins to the 26S proteasome for degradation (Chau et al., 1989; Hershko and Ciechanover, 1998; Thrower et al., 2000; Lu et al., 2015) **Other** • Involved with Wnt signaling propagation (Tauriello and Maurice, 2010) • Indirectly regulates protein activity by signaling the degradation of various inhibitors (Winston et al., 1999; Margottin-Goguet et al., 2003) • Can impair protein interactions without triggering degradation (Flick et al., 2006) • Role in innate immune response signaling (Rajsbaum et al., 2014; Hage and Rajsbaum, 2019)	• Predominant linkage type in cells, often >50% of all linkages (Xu et al., 2009b; Dammer et al., 2011; Kim et al., 2011; Wagner et al., 2011; Ziv et al., 2011) • Levels increase upon proteasome inhibition (Xu et al., 2009b) • Increases in response to mitochondrial depolarization (Ordureau et al., 2014)	**E2** • **Ube2K** (Petroski and Deshaies, 2005; Christensen et al., 2007; Kim et al., 2007; Rodrigo-Brenni et al., 2010; Rajsbaum et al., 2014) • **Ube2G2** (Li et al., 2007) • **Ubc1** (Petroski and Deshaies, 2005; Christensen et al., 2007; Rodrigo-Brenni et al., 2010) • **Ube2R1/Cdc34** (Petroski and Deshaies, 2005; Li et al., 2007; Rodrigo-Brenni et al., 2010; Sadowski et al., 2010) • **Ube2D** (Wang and Pickart, 2005; Kim and Huibregtse, 2009) **E3** • BRCA1/BARD1 (Christensen et al., 2007; Kim et al., 2007) • SCF (Petroski and Deshaies, 2005) • AMFR (Chen et al., 2006; Li et al., 2007) • **E6AP** (Scheffner et al., 1993; Wang and Pickart, 2005; Kim and Huibregtse, 2009) • KIAA10/UBE3C (Sloper-Mould et al., 2001) • Bacterial HECT-like E3 (Lin et al., 2011) • Parkin (Doss-Pepe et al., 2005; Geisler et al., 2010) • N1eL (Hospenthal et al., 2013) • AREL1 (Michel et al., 2015) • UFD2 (Saeki et al., 2004) • TRIM6 (Rajsbaum et al., 2014)	• **OTUB1** (Swatek and Komander, 2016) • USP5/IsoT (Reyes-Turcu et al., 2008)
Lys63	**Scaffolding to facilitate protein interactions** • Acts as an interaction point for the formation and activation of various complexes and pathways: • Activation of NF-κB transcription factor (Deng et al., 2000; Wang et al., 2001; Xia et al., 2009; Xu et al., 2009a) • DNA repair (Spence et al., 1995; Hoege et al., 2002; Sobhian et al., 2007; Doil et al., 2009; Huang et al., 2009; Stewart et al., 2009; Al-Hakim et al., 2010) • Innate immune responses (Gack et al., 2007) • Mitophagy (Cunningham et al., 2015; Ordureau et al., 2015) • Protein sorting (Lauwers et al., 2009; Huang et al., 2013) • Assembly of protein complexes that drive mRNA splicing and translation (Spence et al., 2000; Bellare et al., 2008; Song et al., 2010; Silva et al., 2015) • Propagation of Wnt signaling (Tauriello and Maurice, 2010) **Lysosomal degradation** • Targets substrate to the lysosome for degradation (Mukhopadhyay and Riezman, 2007; Raiborg and Stenmark, 2009; Ren and Hurley, 2010) • Serves as an interaction point for adaptor molecules of autophagosomes and substrates *en route* to lysosomal degradation (Kirkin et al., 2009)	• Second most abundant chain type, after Lys48 (Chen and Sun, 2009) • Levels increase in response to mitochondrial depolarization (Ordureau et al., 2014)	**E2** • **Ube2N/Uev1a/Ubc13** (Zhang et al., 2005; Eddins et al., 2006; Christensen et al., 2007; Kim et al., 2007; Xia et al., 2009) • Ube2D (Kim and Huibregtse, 2009; Maspero et al., 2011) **E3** • BRCA1/BARD1 (Christensen et al., 2007; Kim et al., 2007) • CHIP (Zhang et al., 2005) • TRIM5 (Pertel et al., 2011) • TRIM21 (McEwan et al., 2013) • **TRIM25** (Zeng et al., 2010) • **RSP5/Nedd4** (Richly et al., 2005; Kim and Huibregtse, 2009; Maspero et al., 2011) • TRAF6 (Cao et al., 1996; Deng et al., 2000; Xia et al., 2009) • Parkin (Doss-Pepe et al., 2005; Geisler et al., 2010; Cunningham et al., 2015)	• **CYLD** (Zeng et al., 2010; Sato et al., 2015) • AMSH (Swatek and Komander, 2016) • POH1 (Hao et al., 2013)
Met1	**NF-κB signaling** • Modifies the IKK complex subunit NEMO in order to allosterically activate IKK in the NF-κB pathway (Rahighi et al., 2009; Tokunaga et al., 2009; Gerlach et al., 2011; Ikeda et al., 2011; Tokunaga et al., 2011; Damgaard et al., 2012) **Other** • Cytokine signaling • Regulation of interferon production (Inn et al., 2011) • Control of Wnt signaling during blood vessel formation (Rivkin et al., 2013)	• Rapidly synthesized in response to activation of inflammatory signaling cascades (Tokunaga and Iwai, 2009; Gerlach et al., 2011; Ikeda et al., 2011; Tokunaga et al., 2011)	**E2** • Ube2K (Tokunaga and Iwai, 2009; Tokunaga et al., 2009; Ikeda et al., 2011; Tokunaga et al., 2011) **E3** • **LUBAC** (Kirisako et al., 2006; Rahighi et al., 2009; Tokunaga and Iwai, 2009; Tokunaga et al., 2009; Gerlach et al., 2011; Ikeda et al., 2011; Tokunaga et al., 2011)	**CYLD** (Sato et al., 2015) • **OTULIN** (Swatek and Komander, 2016)
Mono-Ub	**Mediates protein interaction** • Can impair protein interactions: • Mono-Ub of Smad4 blocks its association with Smad2 (Dupont et al., 2009) • Blocks interactions of adaptor proteins to cargo in EGFR signaling (Polo et al., 2002; Hoeller et al., 2006, 2007) • Recruits enzymes to specific cellular locations in response to DNA damage (Hoege et al., 2002; Bienko et al., 2005, 2010; Nijman et al., 2005; Huang et al., 2006; Moldovan et al., 2007; Moldovan and D’Andrea, 2009; Freudenthal et al., 2010; Huang and D’Andrea, 2010; Joo et al., 2011): • PCNA, FANCD2, and FANCI are all mono-Ub-d and also involved in DNA repair pathways (Hoege et al., 2002; Bienko et al., 2005, 2010; Nijman et al., 2005; Huang et al., 2006; Moldovan et al., 2007; Moldovan and D’Andrea, 2009; Freudenthal et al., 2010; Huang and D’Andrea, 2010; Joo et al., 2011) **Lysosomal degradation** • Targets substrates to the lysosome for degradation (Mukhopadhyay and Riezman, 2007)	• Reduced in response to proteasome inhibition, most likely in favor of polyubiquitin chain formation (Kaiser et al., 2011) • Levels vary among tissue, cell, and model types. It is the most abundant, conjugated form of ubiquitin and may rival levels of free mono-ubiquitin (Kaiser et al., 2011)	**E2** • Ube2D (Wang et al., 2004; Bentley et al., 2011) • UbcH5 (Wang et al., 2004; Bentley et al., 2011) • Ube2A (Hoege et al., 2002; Hwang et al., 2010; Hibbert et al., 2011) • Ube2W (Machida et al., 2006; Christensen et al., 2007; Alpi et al., 2008; Scaglione et al., 2011) • Ube2T (Machida et al., 2006; Alpi et al., 2008) **E3** • BMI1-RING1 (Wang et al., 2004; Bentley et al., 2011) • Rad18 (Hoege et al., 2002; Hwang et al., 2010; Hibbert et al., 2011) • FANCL (Machida et al., 2006; Alpi et al., 2008) • BRCA1/BARD1 (Christensen et al., 2007; Scaglione et al., 2011) • CHIP (Christensen et al., 2007; Scaglione et al., 2011) • Parkin (Chew et al., 2011) • CUL3 (Jin et al., 2012; Werner et al., 2015)	• None reported

In addition to proteasomal degradation, mono-Ub and Lys63-linked chains target plasma membrane proteins to lysosomes for degradation (Mukhopadhyay and Riezman, 2007; Chen et al., 2019; Gomez-Diaz and Ikeda, 2019). One type of lysosomal degradation, macro-autophagy (hereafter referred to as autophagy), is induced by cellular stress and is marked by the formation of cytoplasmic, double-membrane vesicles that envelop damaging cellular components, including protein aggregates and damaged organelles. These autophagic vesicles, called autophagosomes, fuse with lysosomes to degrade and recycle their contents. Autophagy involves Ub at several steps. For example, autophagy-inducing factors are regulated by ubiquitination; Lys63 poly-Ub promotes their stability and induces autophagy (Nazio et al., 2013; Xia et al., 2013), while Lys48 poly-Ub targets them to the proteasome and inhibits autophagy (Xia et al., 2014; Xu et al., 2014). Similar ubiquitination events control the genesis and maturation of autophagosomes by promoting the degradation, stabilization, or interactions of various autophagy factors (Joachim et al., 2017; Feng et al., 2019; Gu et al., 2019; Scrivo et al., 2019). Mitophagy, the selective autophagy of damaged mitochondria, is dependent on the E3 Parkin’s Lys6, Lys11, Lys48, and Lys63-linked poly-ubiquitination of the mitochondrial outer membrane (Harper et al., 2018). Although ubiquitination is most commonly associated with the proteasome, its importance to other degradative pathways is also well-established.

Outside of degradation, ubiquitination coordinates the recruitment of various proteins to participate in signaling pathways, alters protein localization by attracting trafficking factors, and even regulates the conformation and activity of the substrate itself (Komander and Rape, 2012; Swatek and Komander, 2016; Oh et al., 2018; Clague et al., 2019). These non-degradation pathway functions are typically, but not exclusively, associated with mono-Ub, Met1-, and Lys63-linked chains (Table 1). Furthermore, chains can induce non-degradative regulation of protein activity indirectly. Met1-, mixed Lys11/Lys63-, Lys63-, and Lys48-linked chains can regulate pathway signaling through inhibitor degradation, allosteric activation as a result of structural reordering, or the recruitment of activating enzymes (Komander and Rape, 2012; Swatek and Komander, 2016; Oh et al., 2018; Clague et al., 2019; Table 1). These various topologies and the nearly limitless types of Ub conjugation that can occur are responsible for the many processes controlled by Ub and the numerous diseases linked to it, from malignancies to neurological diseases and afflictions of the immune system.

Decades of research have been dedicated to the study of ubiquitination, yet it often seems that we have only begun to understand its countless functions in the cell and the players that direct Ub’s roles. What specifies which proteins are ubiquitinated, and with what type of chain? Determinants of substrate specificity include the E2 and E3 enzymes involved (Table 1). A ubiquitination event is initiated when the E1 activating enzyme hydrolyzes ATP and forms a thioester bond with Ub. The Ub is then passed to an E2 conjugase through a transthiolation reaction. An E3 ligase then facilitates the formation of an isopeptide bond between Ub and a lysine in its target protein. How this final step happens depends on the type of E3 involved.

Ub ligases are classified into three families based on constituent domains and Ub transfer mechanisms: RING, HECT, and RBR. RING (Really Interesting New Gene) E3s, the most abundant type, serve as scaffolds to enable the direct transfer of Ub from the E2 to the target protein. HECT (Homologous to the E6AP Carboxyl Terminus) E3s transfer Ub in two steps: a transthiolation event moves the Ub molecule from the E2 to the E3, before passing it to the substrate. RBR (RING-Between-RING) E3s also work in distinct steps: a RING domain recruits a Ub-charged E2, a RING-like domain forms a thioester bond with the Ub, and the Ub is subsequently transferred to the substrate. While the human genome contains only two E1 Ub-activating enzymes, there are ∼40 E2 conjugases and more than 600 E3 ligases that can contribute to substrate and linkage specificity. HECT and RBR E3s each determine the types of Ub linkages they create, while RING E3s depend on their cooperating E2 to impart that specificity (Christensen and Klevit, 2009; Komander and Rape, 2012).

Once a Ub chain has been formed on a substrate, it can be removed or edited by deubiquitinating enzymes (DUBs). Humans have ∼100 DUBs, some of which have preferences for chain type, length, and location, while others are promiscuous in those regards (Table 1). DUBs are divided into seven sub-families: Ub-specific proteases (USPs), Ub C-terminal hydrolases (UCHs), ovarian tumor proteases (OTUs), Machado-Joseph disease proteases (MJDs), JAB1/MPN/MOV34 metalloenzymes (JAMMs), motif interacting with Ub-containing novel DUB family (MINDYs), and zinc-finger with UFM1-specific peptidase domain proteins (ZUFSPs). USPs, UCHs, OTUs, MJDs, MINDYs, and ZUFSPs are cysteine proteases, whereas JAMMs are zinc metalloproteases (Clague et al., 2012, 2019; Abdul Rehman et al., 2016; Kwasna et al., 2018).

The significance of DUBs is evident from the beginning of a Ub molecule’s existence, as Ub genes are transcribed as peptide-linked tandem repeats (*UBB* and *UBC* gene products) or as ribosomal fusion proteins (*UBA52* and *RPS7A* gene products) that require processing by DUBs before they can be used as mono-Ub for substrate ubiquitination (Clague et al., 2012; Komander and Rape, 2012). DUBs also serve as negative regulators of Ub signaling; for example, a protein with a Lys48-linked tetra-Ub chain attached as a proteasomal targeting signal can be spared of degradation by a DUB that removes that chain (Clague et al., 2013, 2019). The 26S proteasome itself contains and is closely associated with DUBs that recognize Ub chains and remove them from degradation-bound proteins to be reused for novel ubiquitination events (Komander et al., 2009; Liu et al., 2015; Clague et al., 2019). Finally, DUBs participate in poly-Ub editing to change the length or composition of the chain, thus modifying the substrate protein’s fate or participation in specific pathways (Komander et al., 2009; Clague et al., 2013, 2019). Some DUBs partner with E3 ligases to attach new Ub molecules, and there is even a DUB that has E3 activity itself, the NF-κB modulator, A20 (Wertz et al., 2004).

DUBs can disassemble chains one molecule at a time [e.g., UCH37 (Lam et al., 1997)], or they can remove the entire chain at once (Figure 1A, bottom left), resulting in unanchored, or free, poly-Ub that is not attached to a substrate protein. An example of the latter type of DUBs, USP14, removes *en bloc* poly-Ub chains from multi-poly-ubiquitinated cyclin B (a cell cycle regulator) until only one chain remains attached, reducing cyclin B’s interaction with the proteasome and thus effectively inhibiting its degradation *in vitro* (Lee et al., 2016). Another DUB, the proteasome resident POH1, is a zinc-dependent metalloprotease that cleaves poly-Ub from substrates, saving the chain from degradation, and yielding unanchored poly-Ub (Yao and Cohen, 2002).

Unanchored Ub chains make their first appearance in the cell at the time of Ub gene transcription, as human *UBB* and *UBC* encode three and nine tandem repeat Ub, respectively. Transcription of these genes results in linear, unanchored poly-Ub that is processed by DUBs that have zinc-finger Ub binding domains (Znf-UBPs) that specifically recognize the chains’ free C-termini, including USP3, USP4, and USP16 (Clague et al., 2019). In mammals, *UBB* and *UBC* transcription is upregulated during cellular stress, when increased signaling requires an ample supply of Ub (Fornace et al., 1989). Unanchored poly-Ub can also be assembled anew by specialized E2/E3 pairs *in vitro* and in cells. This type of production is observed when the E3 TRIM6 and the E2 UbE2K generate unanchored, Lys48-linked Ub chains that activate an interferon (IFN) signaling component (Rajsbaum et al., 2014). Moreover, as mentioned above, *en bloc* removal of a Ub chain from a substrate, or cleavage of a poly-Ub branch from a branched chain, in total or in part, also yields unanchored poly-Ub.

The study of free poly-Ub is relatively new, and we have only begun to understand their complicated nature. They are commonly thought of as potentially toxic competitors at the proteasome, and their rapid disassembly is thought to be essential to cellular health and Ub homeostasis. However, untethered Ub chains are also directly implicated in specific pathways, including NF-κB-related processes; thus, their presence in the cell is clearly important. Yeast and *in vitro* studies suggested that unanchored poly-Ub inhibit normal proteasomal function and are toxic, whereas *Drosophila* studies more recently indicated that they are tolerated in an intact, multi-cellular organism. For the remainder of this review, we examine the details and complexities of unanchored Ub chains.

## Cases of Toxicity From Unanchored Ubiquitin Chains

Much of our knowledge of unanchored poly-Ub arose from research on the best-known DUB that processes it, Ub-specific protease 5 (USP5). Mammalian USP5 (also known as isopeptidase T) was first purified from reticulocytes in 1985 (Pickart and Rose, 1985) and later characterized as an enzyme that preferentially disassembles poly-Ub species after they are removed from ubiquitinated substrates at the 26S proteasome (Hadari et al., 1992). USP5 specifically recognizes the free C-terminal diglycine (GG) motif of untethered poly-Ub and removes Ub monomers sequentially from the chain’s proximal end (Wilkinson et al., 1995). Kinetic assays revealed that Ub, itself, can modulate USP5 activity *in vitro*: low Ub concentrations activate USP5, whereas partial inhibition is observed at higher Ub concentrations (Stein et al., 1995).

USP5 contains four Ub-binding domains that cooperate to recognize and process multiple types of unanchored poly-Ub, with a preference for Lys48-linked chains (Reyes-Turcu et al., 2008). Its ZnF-UBP domain governs USP5’s specificity for untethered chains, with a specialized pocket that recognizes the unencumbered C-terminus of the proximal Ub (Reyes-Turcu et al., 2006). In the fruit fly, *Drosophila melanogaster*, USP5 knockdown or null mutation is developmentally lethal (Tsou et al., 2012; Wang et al., 2014a; Kovacs et al., 2015; Ristic et al., 2016). One proposed purpose for USP5 is to maintain a pool of available mono-Ub by cleaving untethered poly-Ub; when USP5 cannot perform this function, the cell would then lack the building blocks necessary for normal ubiquitination. However, toxicity from RNAi knockdown of USP5 in the fruit fly was not alleviated by over-expression of mono-Ub (Ristic et al., 2016); thus, toxicity arising from reduced or absent activity of USP5 cannot be explained solely by a disruption of mono-Ub supply, and may involve dysregulation of specific ubiquitinated substrates (García-Caballero et al., 2014, 2016).

When Ub is properly folded, several hydrophobic amino acid residues converge to form a hydrophobic surface that serves as the interaction site for many Ub-binding domains (Deveraux et al., 1994; Beal et al., 1996, 1998). It is through this hydrophobic patch that substrate-conjugated poly-Ub binds S5a, a subunit of the regulatory compartment of the 26S proteasome, allowing proteasome-resident and -associated DUBs to detach the chain and enhance the degradation of the targeted protein (Deveraux et al., 1994; Beal et al., 1996). Could unanchored Ub chains directly cause toxicity by binding to the proteasome in lieu of ubiquitinated substrates, interrupting normal proteolysis? Using reconstituted reticulocyte proteasome complexes to study USP5 function *in vitro*, Hadari et al. (1992) determined that USP5 stimulates the proteolysis of poly-ubiquitinated substrates. If unanchored Ub chains can outcompete ubiquitinated substrates at the proteasome and hinder normal proteolysis, the authors suggested that USP5 prevents this by quickly disassembling chains – as soon as they are removed from their substrates – thus enhancing proteolysis by removing competition (Hadari et al., 1992). In this study, the authors did not perform binding assays to assess competition between unanchored chains and ubiquitinated substrates at the proteasome, instead focusing on the effect of USP5 on *in vitro* proteolysis and inferring a corresponding effect from unprocessed poly-Ub.

In follow-up studies, Piotrowski et al. (1997) synthesized Lys48-linked, untethered poly-Ub of various lengths (from Ub^2^ to Ub^8^) and examined their effects on *in vitro* proteasomal function. The authors found that Ub chain length dictates the magnitude of proteasomal inhibition and the chain’s affinity for the proteasome: longer chains are more likely to bind and more strongly inhibit the proteasome (Piotrowski et al., 1997). Later, Thrower et al. (2000) identified Ub^4^ as the minimum signal for efficient proteasomal degradation and showed that unanchored chains compete with ubiquitinated substrates to bind to purified, mammalian proteasomes, again in an *in vitro* setting.

*In vivo* studies in yeast support the notion that unanchored poly-Ub can inhibit proteasomal activity. When Amerik et al. (1997) deleted the *UBP14* gene encoding the USP5 ortholog in *Saccharomyces cerevisiae* cells, they observed accumulation of unanchored Ub chains and inhibition of the Ub-dependent, proteasomal degradation of MATα2, L-βgal, and Ub–P-βgal, reporter proteins used to study Ub-dependent protein turnover. Reasoning that free Ub chains may be the culprits hampering proteasomal degradation, they expressed in wild-type yeast cells a Ub mutant lacking the C-terminal diglycine residues required for Ub’s conjugation onto other proteins and its recognition by UBP14. This mutant (UbΔGG) can be ubiquitinated, allowing for the creation of unanchored, UBP14-resistant poly-Ub with the mutant Ub molecule at the end (Amerik et al., 1997). (Similar mutants that lack an intact C-terminal “GG” motif are used in studies described below; although the precise amino acid mutations vary, the effect is the same, and all such mutants are referred to as “UbΔGG” throughout this review.) Yeast cells expressing UbΔGG mutants suffer growth defects, sensitivity to environmental stressors, and a reduction in overall protein degradation (Ecker et al., 1987; Hodgins et al., 1992). The presence of unanchored, UbΔGG chains coincided with reduced proteolysis of MATα2 and Ub–P-βgal, again indicating proteasomal dysfunction *in vivo*, in a single cellular organism (Amerik et al., 1997).

Moving beyond purified proteasomes and yeast studies, Dayal et al. (2009) linked USP5 to unanchored chain disassembly in a mammalian cellular system. From a screen of DUBs affecting the activity of the tumor suppressor, p53 in cultured, ARN8 human melanoma cells, the authors found that knockdown of USP5 stabilizes and activates p53. They also observed by Western blotting an increase in low molecular weight Ub species that migrate at the same molecular weight as purified, free Lys48-linked chains, and concluded that these are unanchored Ub species induced by USP5 suppression. Expression of UbΔGG recapitulated the effects of USP5 knockdown in ARN8 cells, leading to increased p53 activity. USP5 siRNA or UbΔGG expression also caused increased ubiquitination of p53. Since this increased ubiquitination, which could be a degradation signal, counter-intuitively coincides with slowed p53 turnover, the authors argued that the effects of USP5 suppression on p53 activity are mediated by the accumulation of unanchored Ub chains that outcompete ubiquitinated p53 at the proteasome and extend its half-life (Dayal et al., 2009); the study did not directly examine whether p53 ubiquitination is consistent with a degradation signal, or whether its binding to the proteasome is impaired in the presence of mutant Ub.

Ultimately, various studies (Hadari et al., 1992; Amerik et al., 1997; Piotrowski et al., 1997; Thrower et al., 2000; Dayal et al., 2009) on unanchored poly-Ub pointed toward a toxic effect, often linked to inhibition of the proteasome and the buildup of proteins that were destined to be degraded. We note that these investigations were performed using *in vitro* systems, a single-cell organism, or cultured mammalian cells; there remains a potential for free Ub chains to behave differently in cells *in vivo* and in multicellular organisms. Ub itself is highly conserved and enzymes that dictate its use, editing, and recycling are also conserved at several levels. But, there is also a significant expansion in the number and types of Ub-related proteins and enzymes with evolutionary progression, leaving open the possibility that free Ub chains may have different roles and be regulated differently among species.

## Unanchored Chains and Their Physiological Roles in Immunity

**FIGURE 2:**
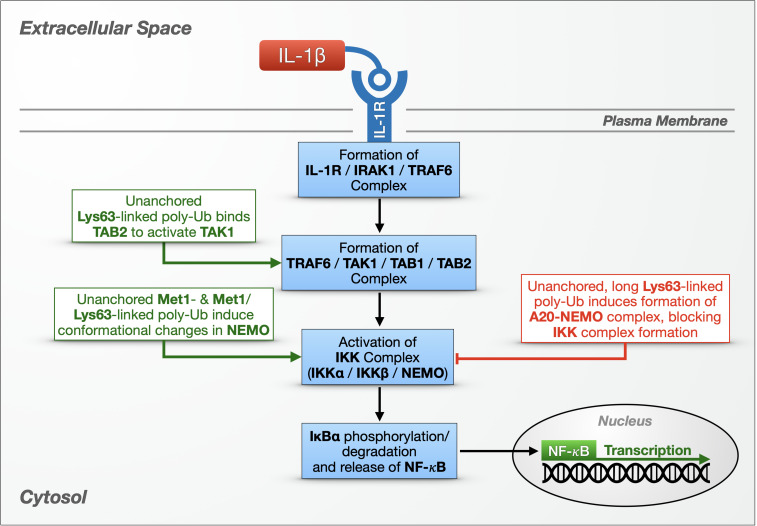
Unanchored poly-ubiquitin in IL-1R signaling. Multiple types of unanchored chains play a role in innate immunity and the NF-κB signaling pathway against bacteria and fungi. In one NF-κB pathway, cytokines – including IL-1β – bind the IL-1 receptor on the cell surface, setting off a cascade that results in the activation and nuclear translocation of NF-κB transcription factors. Additional details are provided in the main text.

**FIGURE 3:**
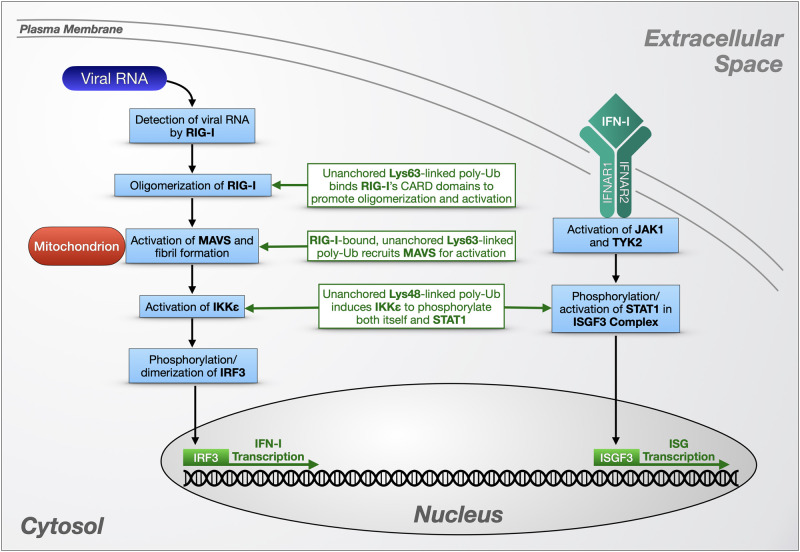
Unanchored poly-ubiquitin in RLR signaling. Unanchored ubiquitin chains play a role in the cellular response to viral RNA via RIG-1 and IFN-I-dependent pathways. RIG-I-like receptors (left portion) detect cytosolic viral RNA and initiate a signaling cascade that triggers the production of antiviral molecules such as interferons (IFNs; right portion) that themselves can initiate additional transcriptional responses to viral presence. Detailed information on these pathways is provided in the main text.

**FIGURE 4:**
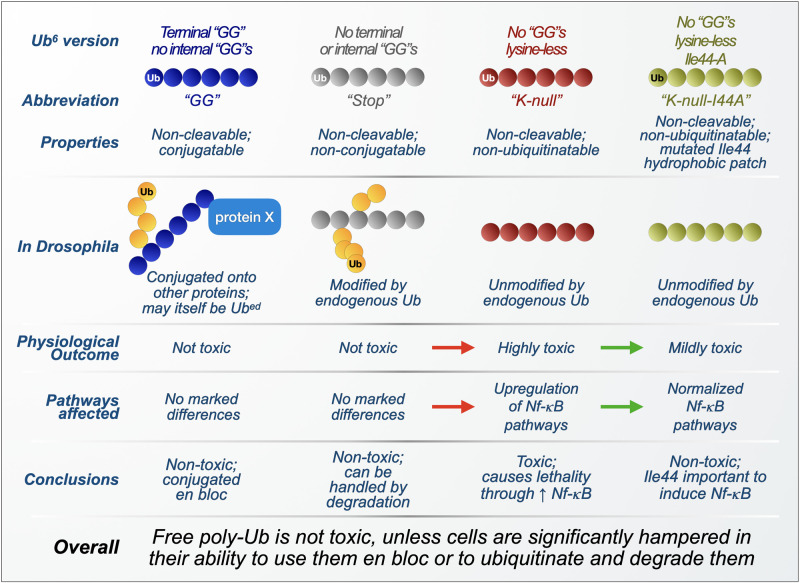
Summary of findings from transgenic, unanchored poly-ubiquitin in *Drosophila*. Overview of the composition, processing, and physiological effects of unanchored ubiquitin chains used in *Drosophila melanogaster*. Red arrows: worsening of phenotypes and outcomes as free poly-ubiquitin is mutated to become resistant to its own ubiquitination. Green arrows: improvement of phenotypes and outcomes as ubiquitination-resistant, free poly-ubiquitin is further modified to inactivate its Ile44 hydrophobic patch. Yellow circles: endogenous ubiquitin proteins.

**FIGURE 5:**
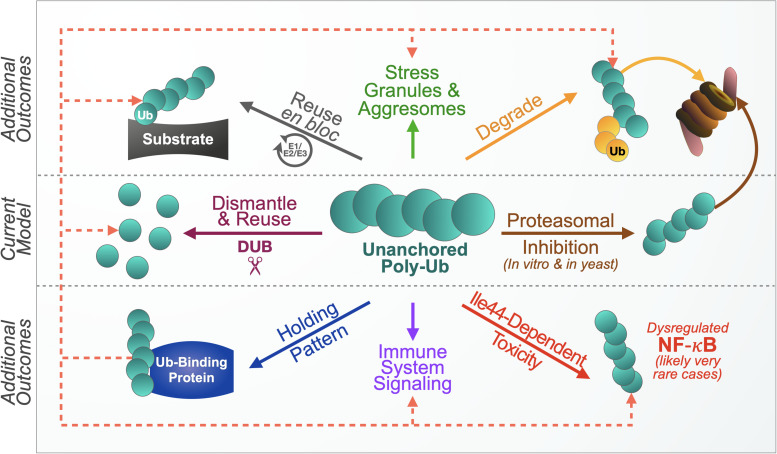
Proposed model of unanchored ubiquitin chain use and control. The current model presents one primary mechanism of handling free chains, their disassembly. Based on increasing evidence, we propose additional outcomes. Free chains can be degraded by the proteasome without inhibiting it, either as they are, or with additional ubiquitination by endogenous ubiquitin (yellow circles). Another mechanism may be achieved through *en bloc* conjugation onto other substrates. Unanchored ubiquitin chains may also associate with ubiquitin-binding proteins in a “reserve” pool for later use in any of the other suggested routes of management (dotted lines). Untethered poly-ubiquitin species also participate in normal immune signaling and response to cellular stress. Lack of control of free chains could lead to adverse outcomes, such as when unanchored poly-ubiquitin that cannot themselves become ubiquitinated activate NF-κB signaling. These possibilities are probably fluidly connected to enable free chain designation among different roles and regulatory routes. We did not depict these intersections in an effort of simplifying the diagram. Additional regulatory mechanisms and functions likely exist.

Beyond their potential for toxicity, untethered Ub chains have been implicated as participants in specific cellular pathways, which we discuss below. Their inclusion in these pathways is evidence that free poly-Ub species are not exclusively toxic, but that they also have important physiological roles in regulating immune pathways that guard against invading pathogens and control inflammatory responses (Figures 2–5).

### TAK1 and IKK Activation

Just as USP5 studies uncovered the potential for untethered poly-Ub to inhibit the proteasome in some systems, much of our knowledge of the normal functions of free Ub chains came to us originally from investigations into Ubc13/Uev1a, an E2 complex that generates unanchored poly-Ub (Xu et al., 2008). Ubc13/Uev1a is important for innate immunity, as it is required for intracellular NF-κB signaling that originates with interleukin-1 receptors (IL-1Rs) and Toll-like receptors (TLRs) (Deng et al., 2000; Wang et al., 2001).

IL-1 cytokines in humans, including IL-1β, are small, proinflammatory proteins that bind IL-1Rs on the cell’s surface (Figure 2). IL-1β binding to IL-1R causes the receptor to form a complex that includes IL-1R-associated kinase 1 (IRAK-1) and the E3 ligase (tumor necrosis factor receptor) TNFR-associated factor 6 (TRAF6). IRAK-1 and TRAF6 dissociate from IL-1R, and TRAF6 joins transforming growth factor β-activated kinase 1 (TAK1), TAK1-binding protein 1 (TAB1) and TAB2 in a new complex in the cytoplasm. Activated TAK1 phosphorylates another cytoplasmic complex, the IκB kinase (IKK) complex, consisting of two catalytic subunits (IKKα and IKKβ) and a regulatory subunit (IKKγ/NF-κB essential modulator, known as NEMO). The IKK complex then phosphorylates IκBα, resulting in its proteasomal degradation and the nuclear translocation of the freed NF-κB proteins (Figure 2; Chen and Chen, 2013; Courtois and Fauvarque, 2018).

Xia et al. (2009) were the first to ascribe a physiological function to unanchored poly-Ub by directly implicating unanchored, Lys63-linked Ub chains in the activation of TAK1 – and, by extension, in the activation of a canonical NF-κB pathway. At the time, it was known that Ubc13/Uev1a is required for TAK1 to phosphorylate the IKK complex (Deng et al., 2000; Wang et al., 2001), but the mechanism was unclear. The authors reconstituted TAK1 activation using purified components and found that unanchored, Lys63-linked poly-Ub generated by Ubc13/Uev1a and TRAF6 triggered the phosphorylation and activation of TAK1 *in vitro* (Xia et al., 2009). Moving to cultured cells, the authors used IL-1β to stimulate HEK-293 cells that stably express IL-1R (termed stable IL-1R cells) and immunoprecipitated the TAK1 complex using TAB2 antibody. The kinase complex co-immunoprecipitated with endogenous, USP5-sensitive (suggesting unanchored) poly-Ub, which they then purified for *in vitro* experiments. The presence of these poly-Ub species activated TAK1 *in vitro* by binding TAB2 at its Npl14 zinc-finger Ub-binding domain, inducing TAK1 phosphorylation. Furthermore, USP5-sensitive poly-Ub species generated by Ubc13/Uev1a and Ubc5, which can make various Ub chain types, beyond Lys63-linked species, activated the IKK complex via NEMO’s Ub-binding domain. The authors proposed a model by which unanchored, Lys63-linked chains bind TAB2 and draw together two TAK1 complexes that mutually phosphorylate and activate one another, while other types of untethered chains bind and activate NEMO in the IKK complex (Figure 2).

What type of poly-Ub activates NEMO has been a complicated question to address. NEMO contains a Ub-binding domain, the Ub binding in ABIN and NEMO (UBAN) domain, that is required for NF-κB activation in mouse embryonic fibroblasts (MEFs) (Rahighi et al., 2009). UBAN can interact with both anchored and unanchored Ub chains, and although it preferentially binds linear poly-Ub with as few as two Ub, longer chains with different linkage types also bind NEMO *in vitro* and in cultured cells (Laplantine et al., 2009; Rahighi et al., 2009; Dynek et al., 2010; Kensche et al., 2012). In stable, IL-1R HEK-293T cells, IL-1β treatment stimulates the production of Lys63-linked poly-Ub decorated with Met1-linked poly-Ub, i.e., branched chains (Emmerich et al., 2013). NEMO can bind these heterotypic chains, based on immunoprecipitation experiments, but it was unclear whether any of the associated poly-Ub species were unanchored. Other *in vitro* studies showed that covalent, Met1-linked di-ubiquitination of NEMO activates the IKK complex more potently than unanchored, Met1-linked Ub^2^, but longer poly-Ub chains were not tested. Spectroscopy studies suggested that NEMO’s interactions with other proteins are mediated by long, linear poly-Ub chains: binding of Met1-linked Ub^10^ induces a conformational change in NEMO that promotes its association with IKKβ and IκBα *in vitro* (Catici et al., 2015). It seems that Ub-dependent NEMO activity can be mediated by both substrate-conjugated and untethered poly-Ub, and the magnitude of the effect may depend on chain length, linkage composition, and the type of interaction or bond.

Untethered Ub chains have also been linked to the negative regulation of NF-κB through their interaction with A20, a dual function enzyme with an N-terminal OTU DUB domain and seven ZnF domains, and with E3 activity at its C-terminus (Wertz et al., 2004). A20 suppresses NF-κB activity in the TNFR and TLR pathways by editing poly-Ub attached to various mediators and by disrupting the assembly of E2/E3 Ub enzyme complexes (Wertz et al., 2004; Shembade et al., 2010). Skaug et al. (2011) discovered an additional, non-catalytic mechanism for A20 suppression of NF-κB activity that depends on unanchored Ub chains. Through *in vitro* experiments including GST pulldowns, Ub-binding assays, and cell-free IKK activation systems, the authors showed that A20 can form a complex with NEMO and long (six or more), untethered, Lys63-linked Ub chains, which then prevents IKK activation. Formation of this complex *in vitro* is aided by long, unanchored chains. Lys63-, Lys48-, and Met1-linked Ub^4^ had no effect; long chains with linkage types other than Lys63 were not tested. Overexpression and RNAi-based studies using HeLa S100 cell extracts confirmed the formation of an A20-NEMO complex, dependent on TRAF6, Ubc13, and A20’s ZnF7 Ub-binding domain. The authors concluded that IL-1β binding to IL1R promotes the assembly of unanchored, Lys63-linked Ub chains by TRAF6 and Ubc13/Uev1a, which then recruit the TAK1 and IKK complexes via Ub-binding domains in TAB2 and NEMO. If A20 is present, it outcompetes TAB2 for poly-Ub binding, and poly-Ub becomes the scaffold in a complex with A20 and NEMO. Formation of this complex inhibits IKK phosphorylation by TAK1, thereby blocking NF-κB signaling (Figure 2).

The studies summarized above (Rahighi et al., 2009; Xia et al., 2009; Skaug et al., 2011; Catici et al., 2015) provide strong evidence that unanchored poly-Ub species contribute to mammalian innate immune responses to bacteria and fungi by modulating at least two steps within NF-κB pathways: (1) the activation of the IKK complex (via TAK1) to phosphorylate IκB and free NF-κB transcription factors, and (2) the termination of TNFR- and TLR-regulated NF-κB signaling by A20 (Figure 2). Collectively, they underscore important roles for free poly-Ub in normal eukaryotic cell physiology.

### RIG-I and IFN-I Pathway Activation

Innate immunity also protects organisms against viruses. A complete virus particle, or a virion, consists of viral DNA or RNA surrounded by a protein shell called a capsid. After entering a host cell, the virion’s capsid is removed and the genetic material is released into the cytosol. Host cell organelles replicate the viral genome and assemble new virions that are released into the extracellular space to infect new cells. Viruses provoke specific immune responses in their hosts, sometimes relying on NF-κB pathways. Human immunodeficiency virus 1 (HIV-1), which causes AIDS, activates NF-κB signaling through TRIM5, an E3 ligase. Interaction with the HIV-1 capsid stimulates TRIM5 to synthesize free Lys63-linked chains that activate TAK1, increasing NF-κB activity to fight the infection (Pertel et al., 2011). Another E3, TRIM21, also produces unanchored Lys63-linked poly-Ub to activate TAK1 in the presence of antibody-bound pathogens in the cytosol (McEwan et al., 2013).

In a separate immune pathway, RIG-I-like receptors (RLRs) detect viral RNA in the cytosol of an infected cell and initiate a signaling cascade that culminates in the production of antiviral molecules including IFNs (Figure 3). Transcription factors involved in this antiviral response include NF-κBs, interferon regulatory factor 3 (IRF3), and IRF7. In contrast to NF-κB proteins, which are activated by the degradation of their inhibitor IκBα, IRF3 and IRF7 are activated by direct phosphorylation by the non-canonical IKKs, IKKε and TANK-binding kinase 1 (TBK1), which causes the transcription factors to dimerize and translocate to the nucleus (Paz et al., 2006; Loo and Gale, 2011).

RIG-I is an RLR expressed at low levels in the cytoplasm of most human cells. When RIG-I detects double-stranded viral RNA, an ATP-dependent dimerization and conformation change is triggered, exposing two tandem, N-terminal caspase activation and recruitment domains (CARDs). These CARDs interact with a CARD on the N-terminus of the signaling adaptor protein mitochondrial activator of virus signaling (MAVS), which in turn activates IKKε and TBK1 to phosphorylate IRF3 and IRF7 (Figure 3; Paz et al., 2006; Loo and Gale, 2011).

To study Ub-dependent mechanisms involved in RIG-I signaling, Zeng et al. (2010) developed a cell-free model of viral infection that combines purified RIG-I protein, mitochondrial and cytosolic extracts, RNA, and ubiquitination enzymes, using IRF3 dimerization as a reporter for RIG-I and MAVS activity. Viral or engineered RNA was used to activate RIG-I in cytosolic extracts, which stimulates MAVS in mitochondrial extracts to promote the dimerization of IRF3. The authors determined that Lys63-linked, unanchored Ub chains are potent, direct activators of RIG-I *in vitro*, binding its tandem CARD domains after RIG-1 detects viral RNA (Figure 3). The authors also verified the presence of this Ub chain type in a human cell line by devising a method to immunoprecipitate endogenous, free poly-Ub from HEK-293T cells using recombinant RIG-I N-terminus [GST-RIG-I(N)]. Some poly-Ub species that bind GST-RIG-I(N) formed β-mercaptoethanol-sensitive thioester bonds with E1 Ub-activating enzyme, indicating a free C-terminus, and were sensitive to both USP5 and the Lys63-specific DUB, CYLD, leading the authors to identify them as unanchored, Lys63-linked chains. The endogenous poly-Ub isolated by this method potently activated IRF3 dimerization in the cell-free system, and their expression was induced by viral infection of HEK-293T cells. TRIM25 is at least partially responsible for producing these Ub chains, as siRNA targeting this E3 ligase diminished Lys63-linked, free poly-Ub levels in HEK-293T cells; conversely, CYLD siRNA led to higher levels, indicating a negative regulatory role for the DUB.

In later studies, Jiang et al. (2012) expanded on these findings by using the reconstituted RIG-I activation assay introduced above to demonstrate that non-covalent binding of free, Lys63-linked Ub chains to RIG-I’s CARD domains promotes its oligomerization (Figure 3). Sedimentation velocity analytical ultracentrifugation indicated that RIG-I specifically forms tetramers in complex with four unanchored poly-Ub; the 4:4 ratio remained constant with all Ub chain lengths tested, from Ub^3^ to Ub^6^. RIG-I formed high molecular weight complexes in response to viral infection in HEK-293T cells and MEFs, but not in Ubc13 knockout MEFs, or in RIG-I knockout MEFs expressing RIG-I that cannot bind Ub, leading the authors to conclude that RIG-I oligomerization depends on binding to Lys63-linked poly-Ub generated by Ubc13. When RIG-I was isolated from both types of knockout MEFs to use in *in vitro* IRF3 dimerization assays, only the higher molecular weight RIG-I aggregates were active, suggesting that Lys63-linked poly-Ub-dependent oligomerization of RIG-I is necessary for its activity. Another RLR with a CARD domain, melanoma differentiation-associated protein 5, behaves similarly to RIG-I *in vitro*, with Lys63-linked Ub^6^ inducing its oligomerization and enhancing its activation of IRF3 in dimerization assays.

Hou et al. (2011) also used the cell-free RIG-I activation system to elucidate MAVS activation. Performing biochemical assays using isolated, crude mitochondrial extracts from virus-infected or uninfected HEK-293T cells, they determined that activated MAVS forms large, prion-like fibrils that induce IRF3 dimerization. MAVS aggregation was induced by RIG-I in the presence of RNA and Lys63-linked, free Ub^4^. Based on these *in vitro* studies and the previous work from Zeng et al., the authors constructed a model for MAVS activation in the viral response that is dependent on binding of unanchored, Lys63-linked poly-Ub to the CARD domains of RIG-I to trigger MAVS aggregation on the mitochondrial membrane (Figure 3; Hou et al., 2011). Crystal structure studies examining covalent and non-covalent binding between RIG-I and poly-Ub revealed that Lys63-linked free Ub^2^ binds RIG-I CARD tetramers, stabilizing them as a scaffold to recruit and activate MAVS to form fibrils *in vitro* (Peisley et al., 2014). Covalent poly-ubiquitination of RIG-I by TRIM25 also induced MAVS aggregation *in vitro*, indicating a potential for multiple Ub-dependent mechanisms to activate this important antiviral pathway. As mentioned above, TRIM25 is a Lys63-specific E3 that also produces at least some of the free poly-Ub chains that activate RIG-I in cultured human cells (Zeng et al., 2010). *In vitro*, the RING domain of TRIM25 partners with distinct E2s like Ubc13/Uev1a or Ubc5 to produce unanchored or substrate-conjugated Ub chains, respectively (Sanchez et al., 2016).

Another TRIM E3, TRIM6, is also involved in viral immunity through RIG-I signaling (Rajsbaum et al., 2014). As mentioned above, IFN IRF3’s phosphorylation and activation are mediated by IKKε (Figure 3). Co-immunoprecipitation experiments using HEK-293T cells and primary human monocyte-derived dendritic cells showed an interaction between TRIM6 and IKKε, and knockdown of TRIM6 depleted IFN-mediated antiviral activity in human lung epithelial A549 cells (Rajsbaum et al., 2014). *In vitro*, IKKε interacted with free, Lys48-linked poly-Ub synthesized by TRIM6 and the E2, UBE2K, and USP5-sensitive poly-Ub interacted non-covalently with IKKε in HEK-293T cells, based on co-immunoprecipitation studies. By confocal microscopy, TRIM6 and IKKε co-localize with Ub-rich, cytoplasmic puncta in HeLa cells, and the formation of these puncta was disrupted by the introduction of USP5, which the authors interpreted to indicate the presence of free poly-Ub in the observed foci (Rajsbaum et al., 2014). *In vitro* phosphorylation assays revealed that untethered, Lys48-linked chains comprising 2 to 16 Ub moieties induce IKKε autophosphorylation, causing IKKε oligomerization and IRF3 activation.

IKKε also contributes to the production of IFN-stimulated genes (ISGs) normally controlled by the JAK-STAT pathway. To activate this pathway, type I IFNs (IFN-I) bind heterodimeric interferon-α/β receptors (IFNARs) on the cell surface, and Janus kinase 1 (JAK1) and Tyrosine kinase 2 (TYK2) phosphorylate signal transducer and activator of transcription 1 (STAT1). Phosphorylated STAT1 forms the IFN-stimulated gene factor 3 (ISGF3) complex with STAT2 and IRF9, which translocates to the nucleus to induce transcription of ISGs (Hage and Rajsbaum, 2019). *In vitro*, IKKε activated by Lys48-linked unanchored poly-Ub directly phosphorylates STAT1 to promote ISGF3 complex formation and increase ISG production (Rajsbaum et al., 2014). Taken together, these studies (Zeng et al., 2010; Hou et al., 2011; Jiang et al., 2012; Rajsbaum et al., 2014; Hage and Rajsbaum, 2019) highlight the importance of two types of unanchored poly-Ub in innate immune pathways (Figure 3).

The participation of free poly-Ub in various immune responses is evidence that these chains have direct, physiological effects that are not limited to the proteasomal toxicity previously seen in yeast and *in vitro* systems (Figures 2, 3). As the pool of research on unanchored Ub chains has grown, there is increasing evidence that chain length and the type of linkage within the chain dictates its specific role in immune signaling [e.g., Lys63-linked chains interact with RIG-I, while Lys48-linked chains activate IKKε (Zeng et al., 2010; Jiang et al., 2012; Rajsbaum et al., 2014)]. The roles of other linkage types remain to be studied in immune signaling and in other physiological processes; thus, it is quite possible that distinct species of free poly-Ub have yet-to-be discovered cellular functions.

## Other Physiological Roles of Unanchored Ubiquitin Chains

Other potential regulatory roles for free poly-Ub species have emerged, beyond their involvement in immune pathways. Braten et al. (2012) expressed a UbΔGG mutant in yeast and performed a gene deletion screen to determine which E2s and E3s are responsible for constructing UbΔGG-terminal chains. By exposing these yeast strains to stressors, they observed that free Ub chains are upregulated during certain types of stress, including heat shock, DNA damage, and oxidative stress, and that the yeast E3, UFD4 generates unanchored chains under basal conditions, while another E3, HUL5 is responsible for most of the ones generated in response to a DNA alkylation agent (Braten et al., 2012). This study did not determine a physiological role for the free chains generated, but their stress-induced upregulation and the identification of two E3s responsible for them is notable and warrants further examination in this model organism and beyond.

Heat shock also induces the reversible formation of cytoplasmic stress granules, which are small, dense aggregations of proteins and mRNA. In cultured HeLa cells, USP5 is recruited to heat shock-induced stress granules, and its knockdown prevents their disassembly, leading Xie et al. (2018) to investigate the potential involvement of unanchored poly-Ub in this process. Heat shocking cells expressing UbΔGG led to the formation of stress granules at the same rate as cells expressing wild-type Ub, but more than twice as many UbΔGG-expressing cells did not clear the newly formed granules (Xie et al., 2018). The authors concluded that untethered poly-Ub interferes with the process of disassembling stress granules; the specifics of this action remain to be determined.

Some cell stressors, including heat shock, can overwhelm or disrupt the proteasome, leading to a buildup of misfolded proteins in the cytosol. When the proteasome cannot meet demand, these proteins form Ub-rich aggregates, which are then amassed into larger inclusions called aggresomes. Aggresomes may be cytoprotective, as they prevent interactions with potentially toxic, misfolded proteins, and they can be eventually cleared via autophagy (Rodriguez-Gonzalez et al., 2008). Ouyang et al. (2012) linked aggresome formation to unanchored poly-Ub associated with misfolded, aggregated proteins. *In vitro* binding assays revealed a direct interaction between the free C-termini of these Ub species and the ZnF-UBP domain of histone deacetylase 6 (HDAC6), which uses the dynein motor complex to transport aggregates to the microtubule organizing center, where aggresomes are formed (Ouyang et al., 2012). Interestingly, proteasomes associate with aggresomes, despite their inability to degrade misfolded protein aggregates within them (Wigley et al., 1999). Based on aggresome clearance assays and immunoprecipitation experiments in cultured HEK-293T cells, unanchored Lys63-linked Ub chains created by *en bloc* cleavage by the proteasomal DUB, POH1 bind to and activate HDAC6, promoting clearance of aggresomes (Hao et al., 2013). In human lung carcinoma cells, the chaperone Hsp90 facilitates the remodeling of aggresome-associated proteasomes, freeing POH1 to efficiently release Lys63-linked unanchored Ub chains that activate HDAC6 and promote autophagic clearance (Nanduri et al., 2015).

Another fascinating aspect of free Ub chains is their counterintuitive contribution to the survival and propagation of viruses within a host organism. Viruses often inhibit immune signaling or exploit normal cellular processes to increase infection, sometimes by directly manipulating unanchored poly-Ub signaling (Hage and Rajsbaum, 2019). Nipah virus, a zoonotic virus that can be fatal in humans, has a matrix protein that antagonizes the RIG-I and IFN-I immune pathways by inhibiting the E3 ligase TRIM6’s production of Lys48-linked unanchored chains; consequently, IKKε is not activated to phosphorylate IRF3 and STAT1, reducing IFN and ISG production (Bharaj et al., 2016). Influenza A viruses (IAVs) use untethered poly-Ub to promote viral uncoating: mimicking misfolded protein aggregates, IAV capsids containing free poly-Ub species recruit HDAC6. Together with cytoskeletal motor proteins, HDAC6 processes the capsid as an aggresome, disassembling it and releasing the viral DNA (Banerjee et al., 2014).

Thus far, not many physiological functions have been ascribed to unanchored poly-Ub; but, it is important to note that they indeed have innate roles and do not appear to simply be byproducts of chain assembly and disassembly. Clearly, additional investigations are needed in intact organisms and in specific cell types and tissues to explore and understand their physiological implications. In the next section, we further explore the idea of free Ub chain toxicity and utilization by discussing outcomes from studies in an intact, multicellular organism, the fruit fly.

## Reduced or Absent Toxicity From Unanchored Poly-Ubiquitin in *Drosophila melanogaster*

The involvement of free poly-Ub in several cellular pathways casts them in a new, more complex light. Given that several types of unanchored chains are now well-characterized as second messengers in immune pathways, it is important to understand their regulation. How do cells maintain optimal levels of the specific types of free poly-Ub they need? When free poly-Ub levels are elevated in an intact organism, is there associated proteasomal inhibition, and furthermore, is that inhibition toxic? What additional signaling roles might there be for untethered Ub chains? In an effort to better understand the physiological functions, regulation, and potential toxicity of unanchored chains, our laboratory conducted a series of studies to determine their impact in an intact, multi-cellular organism. Our collective results provide additional nuance to the emerging understanding of the diversity of unanchored poly-Ub species and their narrow potential for toxicity.

We began our investigations by designing two types of unanchored poly-Ub transgenes to express in *D. melanogaster.* Both types of chains consist of six Ub in tandem and lack internal “GG” motifs that are necessary for isopeptide bond formation and disassembly (Komander et al., 2009; Komander and Rape, 2012; Blount et al., 2018; Clague et al., 2019). Whereas one type of chain also lacks a terminal “GG,” and thus cannot be conjugated onto other proteins (referred to as Ub^6^-Stop), the other chain type contains a “GG” motif at its end to allow for protein conjugation (referred to as Ub^6^-GG; Figure 4). Their expression was enabled by the binary, Gal4-UAS system, which allows for tissue-specific as well as timed expression through specific “drivers” and compounds delivered in fly media (Brand and Perrimon, 1993; Brand et al., 1994; Osterwalder et al., 2001; Roman et al., 2001).

Biochemical analyses of the *Drosophila* lines generated with these transgenic free Ub chains showed robust expression and ample modification by endogenous Ub, effectively creating branched Ub chains with various linkage combinations (Blount et al., 2018). When expressed in all cells, Ub^6^ did not impact *Drosophila* development or adult fly longevity under both normal and heat-stressed conditions. Specific expression in several tissues had moderate effects on longevity, with glial and neuronal expression reducing fly lifespan by a few days (Blount et al., 2018). Overall, longevity results indicated that unanchored chains are not necessarily toxic.

Because of studies indicating an inhibitory role for unanchored poly-Ub at the proteasome *in vitro* and in yeast, proteasomal activity was assessed in flies expressing, or not, Ub^6^-Stop in all cells. Expression of Ub^6^-Stop (which cannot itself be conjugated onto other proteins and thus remains unanchored) did not affect protein levels of proteasomal components, nor did it hinder the turnover of known proteasomal substrates (Blount et al., 2018), suggesting that these linear Ub species do not affect proteasomal function in the manners observed with certain free chains *in vitro* and in yeast. Genetic and biochemical studies indicated that Ub^6^-Stop is itself degraded by the proteasome, highlighting this mechanism as a key regulator of the stability of linear, unanchored poly-Ub in the fly.

Generally, ubiquitination is considered to occur via serial addition of mono-Ub onto an extending chain or substrate. Intriguingly, the conjugation-capable Ub^6^-GG could be attached as a single unit onto other proteins in flies and in cultured, HEK-293 mammalian cells (Blount et al., 2018). These results suggest that Ub recycling need not only occur at the level of a monomer; in fact, a whole chain might be removed from one substrate and attached onto another *en bloc*. While the notion of *en bloc* transfer of an intact chain, without its disassembly into mono-Ub, has been purported before and has been observed *in vitro* (Bamezai et al., 1989; Chen and Pickart, 1990; Li et al., 2007; Masuda et al., 2012), insofar as we know it had not been shown before in a cell or intact animal. These findings led to the possibility of free poly-Ub regulation beyond their disassembly into mono-Ub: they can be degraded by the proteasome, or become conjugated onto other proteins whole-sale, painting a wider tableau of possibilities for unanchored chain regulation in the cell.

Because of the surprising finding that untethered, linear poly-Ub species are not toxic in flies (Blount et al., 2018), we wondered whether *Drosophilae* mount a response toward these chains. We examined changes at the transcriptional level through RNA-seq, using flies expressing everywhere Ub^6^-Stop, Ub^6^-GG, or no transgene with the same genetic background. The presence of each type of Ub^6^ chain led to significant changes in the expression of approximately 90 fly genes, but with no clear, coordinated cellular response to indicate the induction of any particular pathways (Blount et al., 2019). Only 30% of the altered *Drosophila* transcripts observed have assigned gene names, and 27% of the genes had no predicted function, opening up the potential for the future identification and characterization of proteins that interact with untethered poly-Ub. It appears that the expression of unanchored poly-Ub in *Drosophila* translates to minimal transcriptomic or organismal response, judging by the low number of altered transcripts and a lack of specificity for Ub-related pathways in those transcripts that were altered (Blount et al., 2019).

The lack of notable toxicity from linear hexa-Ub chains in *Drosophila* does not dovetail with earlier studies that strongly indicated that unanchored chains toxically inhibit the proteasome in yeast (Hadari et al., 1992; Amerik et al., 1997; Piotrowski et al., 1997; Thrower et al., 2000), leading us to wonder how unique characteristics of unanchored poly-Ub species control their effects. As previously mentioned, Ub^6^-Stop is ubiquitinated in flies, introducing endogenous Ub that could potentially change the way these chains are handled (Blount et al., 2018). To assess the influence of endogenous ubiquitination on the toxicity of unanchored chains, we mutated all lysine residues in Ub^6^-Stop to alanine, creating Ub^6^-Stop-K0, a ubiquitination-resistant, linear hexa-Ub chain that cannot be cleaved by DUBs or conjugated onto a substrate (Figure 4). Mirroring the longevity assays we used to characterize Ub^6^, we observed that Ub^6^-Stop-K0 is significantly more toxic than its ubiquitination-prone counterpart: its expression markedly reduces fly lifespan or is developmentally lethal, depending on expression pattern (Blount et al., 2020). Compared to the ubiquitination-prone Ub^6^, turnover of Ub^6^-Stop-K0 is slowed but not halted, indicating that ubiquitination aids in the turnover of linear Ub^6^, but is not essential to its eventual clearance (Blount et al., 2020).

To explain the enhanced toxicity of ubiquitination-resistant Ub^6^, we turned to the previously described studies linking unanchored poly-Ub to NF-κB pathways (Xia et al., 2009; Rahighi et al., 2009; Zeng et al., 2010; Hou et al., 2011; Skaug et al., 2011; Jiang et al., 2012; Rajsbaum et al., 2014; Catici et al., 2015), reasoning that Ub^6^-Stop-K0 could increase NF-κB signaling (Zhou et al., 2005; Valanne et al., 2011; Chen and Chen, 2013; Myllymaki et al., 2014; Courtois and Fauvarque, 2018). In RNAi studies, knockdown of NF-κB components extends the lifespan of Ub^6^-Stop-K0 flies, although not to the extent of controls (Blount et al., 2020). RT-qPCR revealed that Ub^6^-Stop-K0 expression causes an increase in mRNA levels of several of these NF-κB components (Blount et al., 2020). Based on these results, we concluded that ubiquitination-resistant, linear Ub chains induce aberrant NF-κB signaling, accounting for at least some of its toxicity in flies.

The studies described in section “Unanchored Chains and Their Physiological Roles in Immunity” show direct interaction between unanchored Ub chains and several Ub-binding proteins that specifically recognize Ub’s Ile44-centered hydrophobic patch, including TAB2 and NEMO (Beal et al., 1996, 1998; Sloper-Mould et al., 2001; Dikic et al., 2009; Komander and Rape, 2012). We thus examined the hydrophobic patch’s role in NF-κB-mediated toxicity from Ub^6^-Stop-K0, mutating Ile44 to alanine in each Ub moiety to create the binding-deficient mutant Ub^6^-Stop-K0-Ile44a (Figure 4). These mutations reversed most of the toxicity as well as aberrant NF-κB signaling in flies and also in cultured, HEK-293T cells (Blount et al., 2020). These results indicated a role for free, linear, ubiquitination-resistant chains in NF-κB signaling that depends on an intact Ile44 hydrophobic patch; without Ile44, ubiquitination-resistant Ub^6^ likely is unable to interact with Ub-binding NF-κB components, prohibiting much of its toxicity *in vivo*.

Taken together, these studies (Blount et al., 2018, 2019, 2020) add yet more complexity to the general understanding of unanchored poly-Ub. Clearly, free Ub chains are not always toxic. In flies, linear hexa-Ub chains are well tolerated, as long as they can be ubiquitinated or conjugated onto other proteins. Due to the current unavailability of genetic techniques to stably express in the fly chains of different topologies and linkages, we were restricted to using linear, head-to-tail chains. However, it bears highlighting that these linear chains are quickly and abundantly decorated with endogenous Ub, and are thus transformed into a pool of free Ub species that comprises linear as well as branched chains consisting of M1, K27, K48, and K63 linkages (Blount et al., 2018). Consequently, the findings summarized in this section pertain to various types of free poly-Ub *in vivo* and, with the above caveats in mind, may be extrapolated to apply more widely to other types of chains. Additionally, the fact that all of these Ub^6^ mutants are based on the same backbone – Met1-linked hexa-Ub – but behave differently in *Drosophila* is evidence of the complicated nature of unanchored poly-Ub *in vivo*.

## Perspectives

The studies described in this review provide a complex picture of unanchored poly-Ub handling and function. It is now apparent that the previous understanding of these chains – that they only exist briefly before being disassembled by DUBs to prevent toxicity and allow mono-Ub recycling – is only part of the picture. These members of the Ub family have clear physiological roles and seem to be controlled in ways that have not garnered much attention so far. It appears that untethered Ub chains are regulated through various mechanisms. Not all of the possibilities we present next need to exist and operate at the same time in each cell. They may be cell type- and cell condition-dependent. Also, evolutionary differences may place more weight on some such pathways compared to others.

We propose the following routes of unanchored poly-Ub control and recycling (Figure 5). The first and in all likelihood major route of regulation and recycling remains the disassembly of free chains into mono-Ub so that they can be reused to modify other proteins. A second route may be that of unanchored poly-Ub degradation, either as they are or through additional decoration by endogenous Ub. A third mechanism of free poly-Ub use and control may be their *en bloc* conjugation onto another substrate, essentially removing them from the unanchored population. Additionally, free chains may associate with Ub-binding proteins in a type of “reserve” pool until they can be re-utilized, which may function in conjunction with the other proposed routes of unanchored chain management. In an example of the latter route – and under special conditions where they may not be able to be dispensed of or controlled in other ways – free Ub chains can interact with NF-κB signaling components, causing abnormal signaling and toxicity. Free poly-Ub is clearly involved in normal immune signaling, and also appears to be involved with stress granule clearance. What we present is not an exhaustive tableau of potential outcomes; further studies will likely lead to the discovery of additional functions and cellular responses.

It may seem that more recent studies revealing unanchored Ub’s physiological roles and lack of toxicity contradict earlier studies that characterized free poly-Ub as harmful, but the seeming discordance only highlights the importance of Ub chain type and context. Some of the previous *in vitro* and yeast studies that suggested proteasomal inhibition by untethered poly-Ub focused on Lys48-linked chains (Piotrowski et al., 1997; Thrower et al., 2000; Dayal et al., 2009), while the Ub^6^ chains expressed in *Drosophila* are head-to-tail (Blount et al., 2018, 2019). In principle, it makes sense that unanchored chains that resemble poly-Ub attached to proteasome-targeted proteins – which are often Lys48-linked – could outcompete proteasomal substrates in a manner not observed with linear chains that are not traditionally associated with proteasomal degradation (Piotrowski et al., 1997; Thrower et al., 2000; Komander and Rape, 2012). This out-competition most likely depends on chain abundance compared to endogenous substrates. [We should note here that in the fly linear chains that could become ubiquitinated, including with Lys48 linkages, did not impact proteasome activity and both ubiquitination-capable and -resistant free Ub^6^ were turned over in the fly, with the latter being a little delayed in the earlier stages of its degradation (Blount et al., 2018, 2020)]. Proximity to the proteasome is likely key: *in vitro* proteasome assays allow close association between Ub chains and the proteasome, with few proteins present to interfere, while Ub^6^ expression in transgenic flies does not guarantee such proximity. It is also clear that free poly-Ub toxicity is dependent on post-translational modification of the chain and its interactions with other proteins. Linear Ub^6^ species did not become particularly toxic until they could no longer be used or modified, at which point they were highly toxic in a manner dependent on their ability to interact with other proteins’ Ub-binding domains. Future studies need to take into account post-translational modifications and interactions that affect free poly-Ub function and processing.

It also stands to reason that there could be evolutionary differences between single-cell organisms like yeast, in which free poly-Ub is toxic, and higher order organisms that may have evolved compensatory mechanisms or pathways that utilize these chains. One function for untethered Ub chains that may be conserved between yeast and mammals involves stress granules: in cultured HeLa cells, free poly-Ub interferes with the clearance of stress granules (Xie et al., 2018). Yeast also form stress granules (Wheeler et al., 2017), and they produce free poly-Ub species in response to stressors like heat shock and DNA alkylation (Braten et al., 2012), but an interplay between stress granules and unanchored Ub chains has not been reported in yeast. In contrast to the conservation of stress granules, we described several studies linking unanchored Ub chains to signaling pathways that are not present in yeast (Srinivasan et al., 2010). Unanchored Ub chains can both positively and negatively regulate NF-κB pathways and viral response in multi-cellular organisms (Xia et al., 2009; Zeng et al., 2010; Hou et al., 2011; Skaug et al., 2011; Jiang et al., 2012; Emmerich et al., 2013; Peisley et al., 2014; Rajsbaum et al., 2014; Catici et al., 2015), but yeast do not have *bona fide* NF-κB pathways. Continued studies will be essential to the discovery of additional physiological functions for free poly-Ub that may not be present in single-cell organisms.

## Conclusions and Future Directions

Exciting research opportunities await unanchored Ub chain biology. Different species of free poly-Ub have unique effects; it will be important to distinguish the type of chain responsible for specific actions within cells, and under what circumstances these actions take place. Markers that recognize unanchored poly-Ub with specific linkage types could be developed to aid in the identification of chain types that are upregulated during certain stress responses, or in the mapping of the cellular distribution of different, untethered poly-Ub species. Since poly-Ub can be transferred *en bloc* to substrates *in vivo*, it will be interesting to investigate whether there are situations in which cells may prefer to use pre-formed chains for ubiquitination – e.g., perhaps the increased demand to replenish ATP during exercise (Baker et al., 2010) promotes the use of pre-formed chains as an ATP-preserving method of poly-ubiquitination. Proteomic-based studies alongside genetic and biochemical investigations will be needed to identify E2s and E3s that can transfer Ub chains *en bloc*, and they can provide additional clues about pathways that involve free chains by identifying signaling proteins that bind or interact with them.

The collection of studies summarized here highlights that unanchored poly-Ub species are multi-faceted entities. These prior investigations necessitate further examinations to comprehensively understand the consequences of their presence in the cell and, by extension, to better understand the intricacies of Ub biology and its roles in normal physiology and in disease.

## Author Contributions

JB, SJ, and ST wrote and edited the manuscript. All authors approved the submitted version.

## Conflict of Interest

The authors declare that the research was conducted in the absence of any commercial or financial relationships that could be construed as a potential conflict of interest.

## Abbreviations

CARD: caspase activation and recruitment domain
DUB: deubiquitinating enzyme
E1: Ub activating enzyme
E2: Ub conjugating enzyme
E3: Ub ligase enzyme
HDAC6: histone deacetylase 6
HECT: Homologous to the E6AP Carboxyl Terminus
HIV-1: human immunodeficiency virus 1
IAV: Influenza A virus
IFN: interferon
IFN-I: type I interferon
IKK: IkB kinase
IL-1R: IL-1 receptor
IRAK-1: IL-1R-associated kinase 1
IRF3: interferon regulatory factor 3
IRF7: interferon regulatory factor 7
ISG: interferon-stimulated genes
ISGF3: interferon-stimulated gene factor 3
JAK1: Janus kinase 1
JAMM: JAB1/MPN/MOV34 metalloenzyme
MAVS: mitochondrial activator of virus signaling
MEFs: mouse embryonic fibroblasts
MINDY: motif interacting with Ub-containing novel DUBs family
NEMO: NF-kB essential modulator
OTU: ovarian tumor protease
RBR: RING-Between-RING
RING: Really Interesting New Gene
RLR: RIG-I-like receptor
STAT1: signal transducer and activator of transcription 1
TAB1: TAK1-binding protein 1
TAB2: TAK1-binding protein 2
TAK1: transforming growth factor b-activated kinase 1
TBK1: TANK-binding kinase 1
TLR: Toll like receptor
TNFR: tumor necrosis factor receptor
TRAF6: TNFR-associated factor 6
TYK2: Tyrosine kinase 2
Ub: ubiquitin
UBAN: Ub binding in ABIN and NEMO
UCH: Ub C-terminal hydrolase
USP: Ub-specific protease
ZnF-UBP: zinc-finger Ub-binding domain
ZUFSPs: zinc-finger with UFM1-specific peptidase domain proteins.
